# Not so unique to Primates: The independent adaptive evolution of TRIM5 in Lagomorpha lineage

**DOI:** 10.1371/journal.pone.0226202

**Published:** 2019-12-12

**Authors:** Ana Águeda-Pinto, Ana Lemos de Matos, Ana Pinheiro, Fabiana Neves, Patrícia de Sousa-Pereira, Pedro J. Esteves

**Affiliations:** 1 CIBIO/InBio—Centro de Investigação em Biodiversidade e Recursos Genéticos, Universidade do Porto, Campus Agrário de Vairão, Vairão, Portugal; 2 Departamento de Biologia, Faculdade de Ciências, Universidade do Porto,Porto, Portugal; 3 Center for Immunotherapy, Vaccines, and Virotherapy (CIVV), The Biodesign Institute, Arizona State University, Tempe, Arizona, United States of America; 4 CITS—Centro de Investigação em Tecnologias da Saúde, IPSN, CESPU,Gandra, Portugal; National Institute of Allergy and Infectious Diseases, UNITED STATES

## Abstract

The plethora of restriction factors with the ability to inhibit the replication of retroviruses have been widely studied and genetic hallmarks of evolutionary selective pressures in Primates have been well documented. One example is the tripartite motif-containing protein 5 alpha (TRIM5α), a cytoplasmic factor that restricts retroviral infection in a species-specific fashion. In Lagomorphs, similarly to what has been observed in Primates, the specificity of TRIM5 restriction has been assigned to the PRYSPRY domain. In this study, we present the first insight of an intra-genus variability within the Lagomorpha TRIM5 PRYSPRY domain. Remarkably, and considering just the 32 residue-long v1 region of this domain, the deduced amino acid sequences of Daurian pika (*Ochotona dauurica*) and steppe pika (*O*. *pusilla*) evidenced a high divergence when compared to the remaining *Ochotona* species, presenting values of 44% and 66% of amino acid differences, respectively. The same evolutionary pattern was also observed when comparing the v1 region of two *Sylvilagus* species members (47% divergence). However, and unexpectedly, the PRYSPRY domain of *Lepus* species exhibited a great conservation. Our results show a high level of variation in the PRYSPRY domain of Lagomorpha species that belong to the same genus. This suggests that, throughout evolution, the Lagomorpha TRIM5 should have been influenced by constant selective pressures, likely as a result of multiple different retroviral infections.

## Introduction

Long periods of co-evolution between retroviruses and their hosts have resulted in the emergence of numerous host defense mechanisms important for an antiviral response, as well as the selection of diverse viral countermeasures [1, 2]. The host attempts to circumvent the viral infection firstly by the activation of innate immune responses initiated by pattern-recognition receptors (PRRs), and secondly by subsequent inducible activation of multiple protective signaling pathways [3, 4]. Restriction factors, sometimes induced by interferon (IFN) signaling, are widely expressed components of the innate host immune system with the ability to inhibit the replication of viruses during their life cycle in host cells [5, 6]. As a result of the selective pressure (positive selection) exerted by viral infections, restriction factors can undergo a rapid evolution of their coding sequences, allowing the host to keep pace with the viral adaptations against these host factors [1, 7]. The study of human innate immune factors responsible for detecting and fighting viral infections has been investigated by comprehensive surveys of the evolutionary relationships between orthologous restriction factor genes in Primates. One such example is the tripartite motif-containing protein 5 alpha (TRIM5α), a cytoplasmic factor that restricts retroviral infection in a species-specific manner [8, 9]. TRIM5α belongs to the TRIM family, characterized by the presence of a RING domain, one or two B-box domains, and a coiled-coil domain [8, 10]. The specificity of Primate TRIM5α restriction has been assigned to the “variable loops” (v1, v2, v3 and v4) of the C-terminal PRYSPRY domain (also known as B30.2 domain), as the sequence identity between species is low and strong evidence of positive selection during Primate evolution has been detected [9, 11].

Given the significance of the Primate TRIM5α evolutionary history, several orthologs have been described for other mammalian species [12–15]. For example, in Lagomorpha a differentiation in the PRYSPRY domain was observed and, even more revealingly, in genera and species with an evolutionary divergence timeline similar to Primates evolution [16]. The order Lagomorpha is divided into two families which diverged around 30–35 million years ago (Mya): Ochotonidae and Leporidae [17]. While Ochotonidae only contains one extant genus, *Ochotona*, the Leporidae family includes 11 genera that comprises *Lepus*, *Sylvilagus* and *Oryctolagus* [18]. TRIM5 of genera *Oryctolagus*, *Sylvilagus* and *Lepus* exhibit an overall similarity between 89% and 91%, but for the variable loop 1 (v1) of the PRYSPRY domain similarity values decrease to 50% [16]. When including *Ochotona*, for the stretch of 30 amino acids in v1 region, 11 have undergone positive selective pressure during Lagomorpha evolution [16]. Such evidence of gene adaptation in Lagomorpha TRIM5 variable loops is indeed very comparable to the selective pressures imposed by exposure to retroviral capsid (CA) in primate TRIM5 genes.

Due to the absence of known infecting exogenous retroviruses in Lagomorpha species, the discovery of the rabbit endogenous lentivirus type K (RELIK) in the genome of several Leporidae genera raised the hypothesis that this retroviral relic could have played a role in the selection of the leporid TRIM5 [19, 20]. Nevertheless, the 12 million year old RELIK is apparently absent in members of the other Lagomorpha family, the Ochotonidae [20]. Recently, the ability of the Lagomorpha TRIM5 to restrict a wide range of retroviruses, and particularly viral vectors containing the RELIK CA, have been examined and supported the previous suggestion of RELIK exerting selective pressures on leporid TRIM5 [21, 22].

The coincident evolutionary divergence aspects between Lagomorpha and Primates orders, and the sequence variation in PRYSPRY domain between different genera of Lagomorpha [16,18], propelled us to conduct a more exhaustive study of the PRYSPRY domain throughout Lagomorpha evolution. While in the extensively studied Primates the PRYSPRY domain sequence and length variations occurred mainly between genera (implying high divergence times) [23–25], we observed an array of intra-genera differences in Lagomorpha. These results provide evidence of different and contemporary selection events throughout Lagomorpha speciation.

## Results

**Fig 1:**
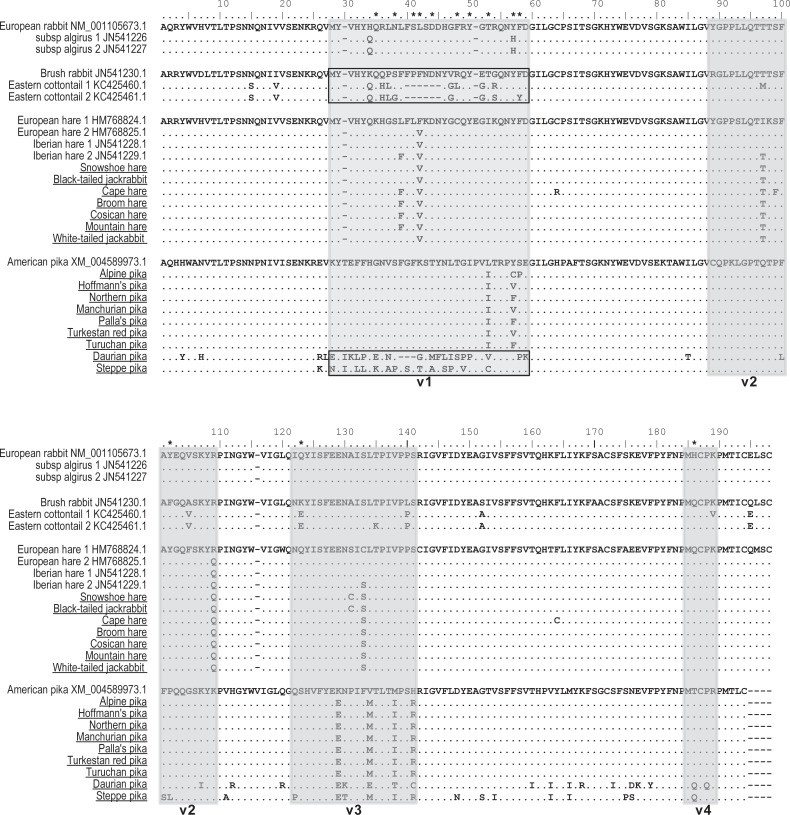
Lagomorpha PRYSPRY sequences. PRYSPRY sequences from 23 Lagomorphs’ genomes, two belonging to the *Oryctolagus* genus, two from *Sylvilagus* genus, nine belonging to the *Lepus* genus and ten to the *Ochotona* genus. Additional members of the *Lepus* and *Ochotona* genera used in this study are underlined. Variable loops from the PRYSPRY domain (v1, v2, v3 and v4) are defined by grey boxes. High variable residues are shown in black boxes. Positively-selected codon positions identified at least by three ML methods are indicated by an asterisk (*).

**Fig 2:**
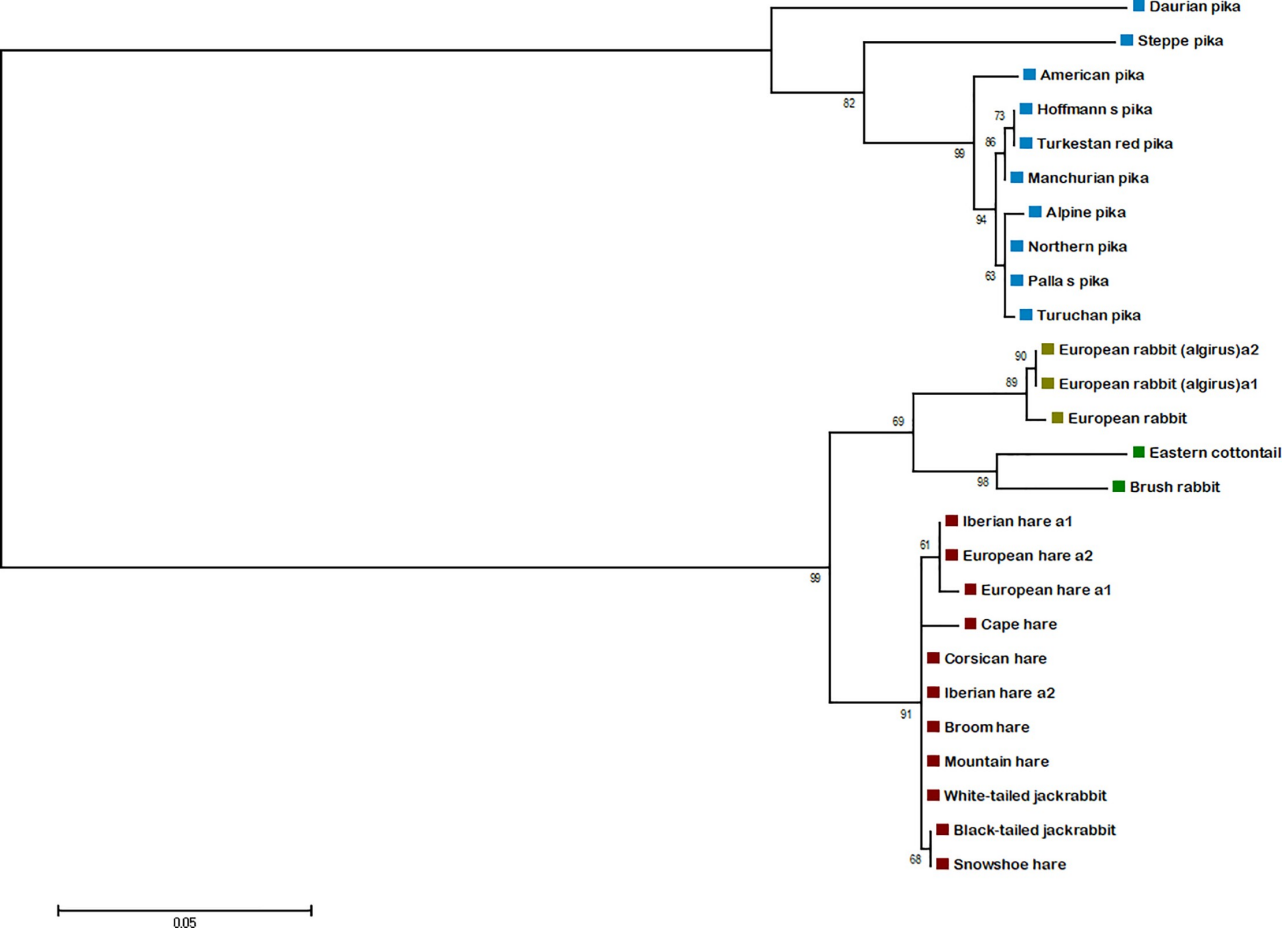
Phylogenetic analysis of PRYSPRY domain of Lagomorpha. Nucleotide sequences of the PRYSPRY domain of Lagomorpha species were used to construct a Maximum Likelihood tree using PhyML v3.0 [27]. The support of the resulting nodes was estimated using 1000 bootstrap replicates. The bootstraps values are indicated on the branches. Blue squares: *Ochotona* species; yellow squares: *Oryctolagus* species; green squares: *Sylvilagus* species; red squares: *Lepus* species.

It is known that the PRYSPRY domain is primarily responsible for the direct binding of TRIM5α protein to retrovirus capsids, leading to structural damage of the incoming viral core and disruption of virus infection [26]. The sequence of *TRIM5* gene has been already obtained in a limited number of Lagomorpha species, namely the European rabbit (*Oryctolagus cuniculus*), the European brown hare (*Lepus europaeus*), the Iberian hare (*L*. *granatensis*), the brush rabbit (*Sylvilagus bachmani*) and the eastern cottontail (*S*. *floridanus*) [16, 22]. Therefore, in this study, we performed a more extensive intra-genus study of the orthologous Leporidae and Ochotonidae PRYSPRY domains, increasing the number of sequences from additional members of *Lepus* and *Ochotona* genera. Here, we present the PRYSPRY domain sequences of seven other *Lepus* species, and also provide the first insight into the variability of *TRIM5* PRYSPRY domain of nine pika (*Ochotona*) species, all of them representative of a wide geographic distribution. In Fig 1 all the deduced PRYSPRY sequences from *Lepus* and *Ochotona* species, along with other Lagomorpha *TRIM5* sequences that have been previously published [16, 22] are represented. The phylogenetic tree generated from the Lagomorpha sequences was supported by high bootstrap values (Fig 2) and the phylogenetic relationships match the species tree [18]; however, Daurian pika and steppe pika present longer branches comparatively to the remaining species (Fig 2).

### Identification of positively selected sites in Lagomorpha PRYSPRY domain

To look for evidence of potential selection pressures acting on the PRYSPRY domain, we used the dataset of Lagomorpha sequences mentioned above and implemented a maximum-likelihood (ML) approach, by using PAML and Datamonkey softwares (see methods for more information). For most protein-coding genes, the rate between nonsynonymous and synonymous substitutions (dN/dS) is a measure of natural selection, with positive selection (dN/dS > 1) acting against the common genotype [28–30]. Comparison of the *TRIM5α* gene dN/dS ratio of several Primates has previously revealed strong evidence of positive selection acting in different species groups (i.e. New World Monkeys, Old World Monkeys and Hominoids), more specifically in the PRYSPRY domain [9]. Similar results were also observed for three leporid genera, where among the 25 positively-selected codons identified for the *TRIM5* gene, 20 were located in the PRYSPRY domain [16]. In this study, we were able to locate 13 sites that reflect strong positive selection pressure in the Lagomorpha’s PRYSPRY domain (S1 Appendix). Among the 13 individual sites detected, only 5 overlap with the previous work [16], a discrepancy resulting from the addition of a greater number of species to *Lepus* and *Ochotona* genera. As seen in Fig 1, residues identified as being under positive selection are within the variable loops v1-v4 (residues under positive selection are marked with asterisk “*” in the alignment). Most importantly, 10 of the 13 residues fall within the v1 variable region, known to be a key determinant domain for virus specificity of TRIM5α restriction activity in Primates [31, 32]. In addition to the strong positive selection found in the v1 region of PRYSPRY domain, this domain has undergone an unusual number of indels. In fact, a deletion of eight predicted amino acid residues has occurred in the eastern cottontail genome, whereas the orthologous v1 region of the European rabbit and *Lepus* genus TRIM5 sequences encode a protein two and one amino acids, respectively, shorter than the American pika sequence.

**Fig 3:**
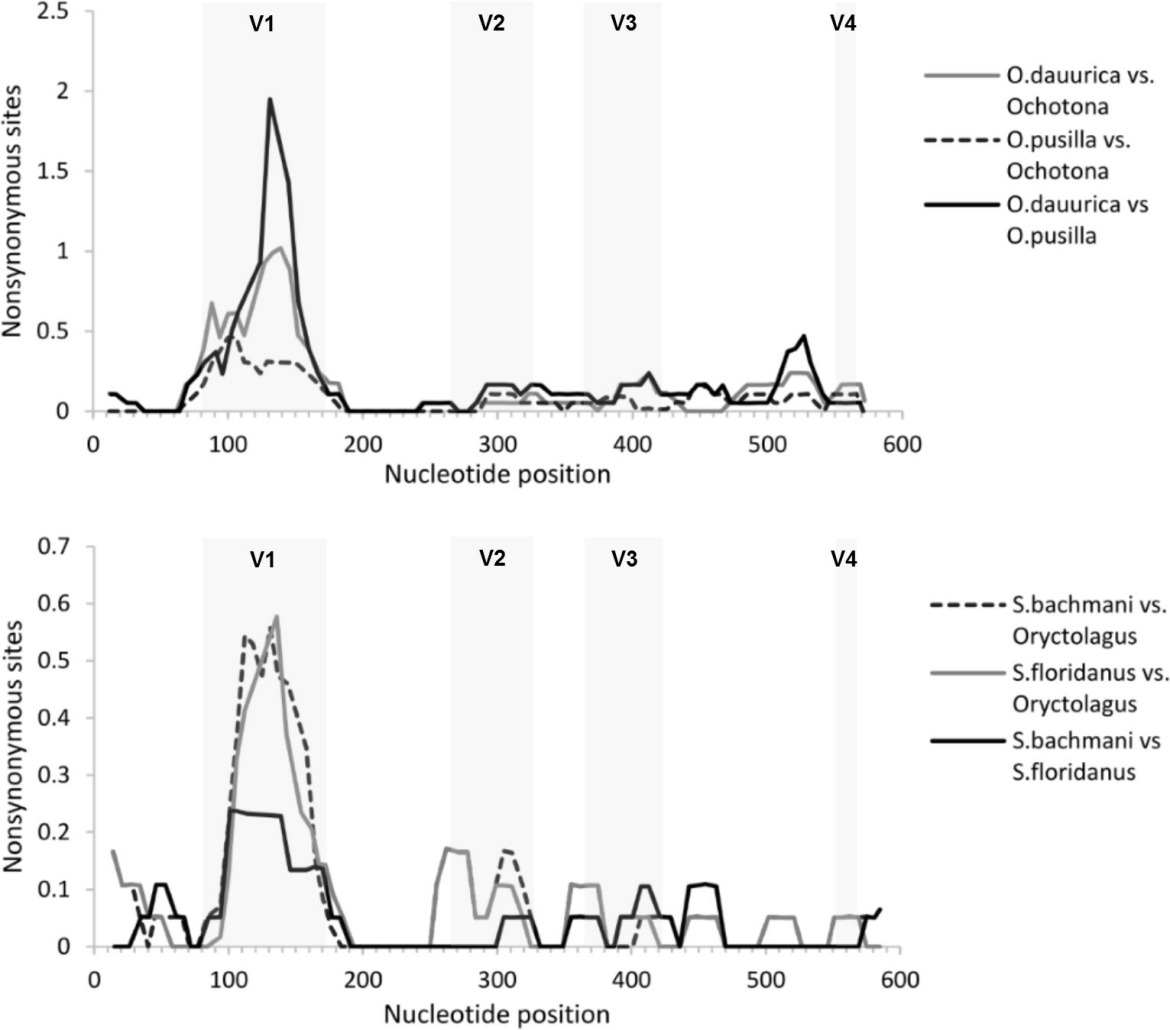
Sliding-window analysis to detect nucleotide differences between PRYSPRY domains from different Lagomorphs. Representation of the non-synonymous per non-synonymous sites between *Sylvilagus bachmani* vs *Oryctolagus genus*, *S*. *floridanus* vs *Oryctolagus* genus and *S*. *bachmani* vs *S*. *floridanus*; *Ochotona dauurica* vs Ochotonidae family, *O*. *pusilla* vs Ochotonidae family and *O*. *dauurica* vs *O*. *pusilla*.

Despite the several modifications that can be observed between different Lagomorpha genera, major differences can also be observed within each genus. Indeed, the deduced amino acid differences between the two *Sylvilagus* species were very dramatic: for example, when comparing the v1 region between the eastern cottontail rabbit and the brush rabbit, the two species registered a remarkable 47% divergence, resulting from the substitution of eight amino acid residues and a deletion of six residues (considered a significant “difference” between sequences) in the eastern cottontail rabbit genome (Table 1). Moreover, when the number of non-synonymous substitutions that gave rise to non-synonymous sites was plotted in a sliding window, this value was undoubtedly higher in the v1 region when compared with the remaining regions of the PRYSPRY domain (Fig 3). Considering the *Ochotona* genus, this domain is quite conserved amongst the different species, with the clear exception of the Daurian pika and the steppe pika (Fig 1). The PRYSPRY variable regions of these two pika species evidenced a high divergence when compared to the remaining *Ochotona*, with special incidence in the 32 residue-long v1 region, showing values of 44% (steppe pika) and 66% (Daurian pika, including the 3 amino acid residues deletion) of amino acid differences (Table 1). Interestingly, the divergence between these two species in this particular region was also extremely high (62%). For the same species, the estimated dN/dS values of the PRYSPRY v1 region showed evidence of positive selection acting on this domain (Table 1). Also, the sliding window in Fig 3 shows that in the v1 region the number of non-synonymous sites was higher than for the remaining regions of the PRYSPRY domain.

**Table 1:** Pairwise estimation of non-synonymous to synonymous substitution ratios (dN/dS) for the PRYSPRY v1 region by using Nei-Gojobori (Jukes-Cantor correction) method [33].

PRYSPRY v1 regions^a^ for comparison	% aa difference^b^	d_N_/d_S_^c^
Brush rabbit vs European rabbit	50 (15/30)	1,96
Eastern cottontail 2 vs European rabbit	50 (15/30)	3,82
Brush rabbit vs eastern cottontail 2	47 (14/30)	0.40
Daurian pika vs American pika	66 (21/32)	5,07
Steppe pika vs American pika	44 (14/32)	3,81
Daurian pika vs steppe pika	62 (20/32)	3,85

^a^ Lagomorpha species PRYSPRY v1 region is indicated in Fig 1.

^b^ The percentages of amino acid residues that differ between the PRYSPRY v1 region of the indicated species are shown. The percentages were calculated as follows: number of different residues/total number of residues compared x 100%. Indels in one sequence were counted as differences and also contributed to the total number of residues compared.

^c^ Pairwise estimation of non-synonymous to synonymous substitution ratios (d_N_/d_S_) using Nei-Gojobori (Jukes-Cantor correction) method.

### Evolution of TRIM5α PRYSPRY domain throughout Primate lineages

To trace whether Primates also manifest a high intra-genus variability within their orthologous PRYSPRY domains, we collected and aligned the PRYSPRY domain coding sequences from 54 Primate species (S2 Appendix), representing ~45 million years of the evolutionary history of Primates [34]. Only the sequences of genera with two or more species were collected, allowing us to query TRIM5α Primates intra-genera differences (S3 Appendix). The translated nucleotide sequences were aligned against *Homo sapiens* PRYSPRY domain and are represented in S2 Appendix.

New World Primates (Platyrrhini) diverged from a common ancestor with Catarrhini about 45 Mya [34]. We were able to obtain 24 sequences of Platyrrhini species, representative of 9 different genera: *Callithrix* (three species), *Mico* (three species), *Leontopithecus* (two species), *Saguinus* (five species), *Aotus* (two species), *Saimiri* (three species), *Alouatta* (two species), *Pitheca* (two species) and *Callicebus* (two species). From the obtained alignment (S2 Appendix), and according to previous reports [23], it is possible to observe that the PRYSPRY domain from Platyrrhini species is very different from the human PRYSPRY domain. In fact, the Platyrrhini species v1 loop is nine amino acid shorter than the v1 loop of *H*. *sapiens*. Of particular note, the two species of *Alouatta* sp. possess an indel of 62 amino acids in the PRYSPRY domain, a feature that is not observed in any other Platyrrhini species.

Catarrhini group includes Cercopithecoidea (Old World Monkeys) and Hominoidea (human, great apes and gibbons). Cercopithecoidea comprises two big subfamilies, Colobinae and Cercopithecinae, with divergence times of ~18 Mya [34]. For Colobinae, we were only able to collect two PRYSPRY sequences, both for *Rhinopithecus* genus. Regarding Cercopithecinae, 19 sequences were obtained, belonging to five different genera: *Cercopithecus* (four species), *Chlorocebus* (four species), *Cercocebus* (two species), *Papio* (three species) and *Macaca* (six species). Interestingly, all *Chlorocebus* species have a longer PRYSPRY domain, as a result of a tandem duplication (S2 Appendix). For Hominoidea, which radiation is approximately 13 Million years old [34], sequences from *Pan* (two species), *Pongo* (two species), *Hylobates* (three species) and *Nomascus* (two species) were obtained.

Consistent with previous reports [23, 35], high variation in the PRYSPRY domain among different families of Primates was observed. However, as readily observed in the aligned sequences (S2 Appendix), and particularly in the highlighted PRYSPRY v1 region, the divergence between Primate species in the same genus was nearly nonexistent, suggesting that much of the evolutionary pressures on this restriction factor were exerted prior to intra-genus speciation.

## Discussion

Under normal circumstances, the host TRIM5α protein binds to the retrovirus capsid (CA) protein via its PRYSPRY domain, blocking the retrovirus replication early in its life cycle [36]. It is now established that species variation on the PRYSPRY domain led to differences in their ability to restrict retroviruses and, therefore, PRYSPRY sequence determines which retrovirus a specific TRIM5α will restrict. For example, human TRIM5α is not effective against human immunodeficiency virus (HIV-1), while TRIM5α proteins encoded by rhesus macaque are able to efficiently restrict this lentivirus [23, 36]. In the light of the extensively described role of TRIM5α PRYSPRY domain in Primates [37–40], previous studies have also supported the same role of this domain in Lagomorpha species. In fact, active TRIM5 proteins were identified and described in several lagomorphs, including the European rabbit, European brown hare, cottontail rabbit and American pika [21, 22]. Evolutionary analysis also showed that TRIM5α has evolved under positive selection in Primates and that this selection has been directed to the PRYSPRY domain [9, 21]. Accordingly, our results reported here reveal that the Lagomorpha PRYSPRY domain is under strong positive selection, with 10 of the 13 positive sites falling in the v1 variable loop (Fig 1). Moreover, our observations were reinforced by the sliding-window analysis of PRYSPRY nucleotide divergence, especially when considering *Ochotona* and *Sylvilagus* species (Fig 3).

Our results reveal that the nine studied *Lepus* species exhibit a very conserved PRYSPRY domain (Fig 1), a relevant finding since *Lepus* is the most geographically distributed genus of all Lagomorpha and some variability on the domain was expected [18]. Fossil records along with phylogenetic approaches suggest that North America is the *Lepus* region of origin [18]. The global development of temperate grasslands (7 to 5 Mya) and the formation of the west Antarctic ice sheet (6.5 Mya) enabled the development of land bridges and consequent rapid expansion and radiation of the *Lepus* genus through Eurasia and into Africa [18]. However, even when using species with different dispersal times and representative of American, European, Asian and African continents, *Lepus* spp. PRYSPRY domain apparently did not undergo strong selective pressures.

A different evolutionary history is reflected by the *Sylvilagus* TRIM5 PRYSPRY domain (Fig 1). The two *Sylvilagus* species included in this study, the brush rabbit and the eastern cottontail, have a divergence time of ~5 Mya and, yet, they present a dramatic divergence on the PRYSPRY v1 region. Interestingly, it appears that the divergence within this domain causes slightly different restriction phenotypes when compared to the TRIM5 PRYPSPRY domains of the remaining Leporids. Murine leukemia viruses (MLV, gammaretroviruses) are generally insensitive to Leporid’s TRIM5 action [21, 22]. However, it is striking that the TRIM5 of the eastern cottontail rabbit is able to restrict two strains of MLV (N-MLV and B-MLV) and partially restrict a third one, Mo-MLV [22]. No known exogenous infecting retroviruses have been described specifically for *Sylvilagus* genus, yet such striking evidence of acting selection on the genus’ TRIM5 probably reflects the action of species-specific exogenous retroviruses still to be found or possibly recently extinct.

Similar to the pattern observed for *Sylvilagus*, the PRYSPRY domain of the ten *Ochotona* species included in this study is also a striking example of ongoing diversification. Eight of the studied species have a conserved TRIM5 PRYSPRY domain (Fig 2). However, the striking evidence of diversification in Daurian pika and steppe pika PRYSPRY domain, not only on the v1 region but throughout the full domain, highly supports the relatively recent existence of species-specific infecting retroviruses.

Recent studies identified the v1 loop as being the most important region of the PRYSPRY domain for the retroviral CA interaction. In humans, it was described that v1 loop has critical residues that allow different conformational changes responsible for the adaptability of this protein to the varying curvatures of retrovirus CA [31, 41, 42]. Studies on Rhesus monkey PRYSPRY domain showed that v2-v3 loops are located in the SPRY subdomain, while v1 loop residues locate in the PRY subdomain [26]. These findings prompted Yang and collaborators (2012) to suggest that the acquisition of PRY domain by the more ancient SPRY domain might be correlated with the emergence of the viral capsid-sensing capacity in vertebrates [26]. The maintenance of several polymorphisms on the variable loops that are responsible for the control of antiviral specificity suggests that different selective pressures may be acting on the PRYSPRY domain of different Lagomorpha species.

To the best of our knowledge, the observed variation within the PRYSPRY domain between closely related species of the same genus was never reported before. Primates have divergence times similar to those of lagomorphs [18, 34]. However, when considering the PRYSPRY domain, we did not find high variations in the amino acid sequences within the different Primates genera (S2 Appendix). Both the *Sylvilagus* and *Macaca* genera diversification began at around the same divergence time (~5 Mya) [18, 34]. However, in contradiction to what was observed for *Sylvilagus* spp., the six *Macaca* species did not present marked amino acid variation in the PRYSPRY domain, with the only difference between species being a two amino acid deletion in *M*. *fascicularis*, *M*. *nigra* and *M*. *thibetana*. The same comparison can also be made between *Ochotona* and *Saguinus* genera, since the diversification of both began at ~8 Mya [17, 34]. In contradiction to what was observed in the Daurian pika and the steppe pika, none of the six *Saguinus* species displayed marked differences in the amino acid sequence of the PRYSPRY domain, especially considering the v1 loop.

So, what is driving the evolution of PRYSPRY domain in Lagomorpha? Initial studies suggested that leporid retroviruses like RELIK, the first reported endogenous lentivirus ever [43], may have imposed positive selection on TRIM5 orthologs. RELIK was first identified in the genome of the European rabbit and, subsequently, in the genome of other leporid genera, including *Lepus*, *Sylvilagus* and *Bunolagus*, which places its origins around 12 Mya [20]. Indeed, the role of endogenous retroviruses, such as RELIK, in driving the evolution of TRIM5 might be determinant, for example, in *Sylvilagus* species; yet, it is apparently less important for the evolution of *Lepus* TRIM5, since the nine *Lepus* species exhibited a great conservation of the PRYSPRY domain (Fig 1).

Retroviruses have been infecting vertebrate hosts for millions of years by integrating their proviral DNA copies as permanent insertions of host genomes. After becoming fixed in a population, endogenous retroviruses (ERVs) can be used as “fossils”, providing a remarkable record of virus-host interactions [44, 45]. In the past years, taking advantage of the European rabbit (oryCun2.0) and American pika (ochPri3.0) genomes assembly, several efforts have been made to detect the presence of other retroviruses, rather than RELIK, in lagomorph’s genomes. For example, a study focused on retroviral diversity across wide samples of vertebrates showed that in European rabbit and American pika the ERVs abundance is dominated by two major groups: Gamma-like ERVs and Beta-like ERVs [46, 47]. A Pika-BERV (pika endogenous betaretrovirus) with an endogenization event calculated ~3–6 Mya is, interestingly, only present in a few *Ochotona* species, including Hoffmann’s pika, Manchurian pika, Alpine pika, Turuchan pika and American pika [48], all part of the alpine group that diverged around 3–6 Mya from the remaining pika groups [17]. In fact, the presence of Pika-BERV in only five *Ochotona* species, representative of a 3–6 million years divergence, reinforces our hypothesis that a primitive retroviral infection could have shaped the PRYSPRY domain of the Daurian pika and the steppe pika. However, taking in consideration that a large number of mammalian retrovirus remain to be identified [46, 47] and that maybe past retroviruses were not endogenized in the Lagomorpha genomes, it is impossible to predict what shaped the TRIM5 evolution in some Lagomorpha species.

Overall, our results fuel the hypothesis that the Lagomorpha TRIM5 evolution might have been impacted by different unknown ancient retroviruses, either endogenous or exogenous. With this work, we demonstrate the multitude of evolutionary independent episodes of TRIM5 variation in different Lagomorpha genera, through the exhibition of species-specific length and sequence variation in the PRYSPRY domain. Such findings were not observed in TRIM5 Primates genera, supporting the uniqueness of TRIM5 Lagomorpha evolution at the species level.

## Materials and methods

### Lagomorpha genomic DNA (gDNA) sources and tissue extraction

Tissues from *Lepus* and *Ochotona* specimens were used to extract genomic DNA (gDNA). Snowshoe hare (*Lepus americanus*), black-tailed jackrabbit (*L*. *californicus*), Cape hare (*L*. *capensis*), broom hare (*L*. *castroviejoi*), Corsican hare (*L*. *corsicanus*), mountain hare (*L*. *timidus*) and white-tailed jackrabbit (*L*. *townsendii*) samples were supplied by CIBIO/InBIO, Vairão, Portugal. Samples from alpine pika (*Ochotona alpina*), Daurian pika (*O*. *dauurica*), Hoffmann’s pika (O. *hoffmanni*), northern pika (*O*. *hyperborea*), Manchurian pika (*O*. *mantchurica*), Palla’s pika (*O*. *pallasi*), steppe pika (*O*. *pusilla*), Turkestan red pika (*O*. *rutila*) and Turuchan pika (*O*. *turuchanensis*) were kindly provided by the Zoological Museum of Moscow State Lomonosov University, Moscow, Russia. gDNA was extracted using the E.Z.N.A.® Tissue DNA Kit (Omega Bio-Tek, Norcross, GA, USA) according to manufacturer’s instructions.

### Lagomorpha TRIM5 PRYSPRY domain amplification and sequencing

Primers were designed according the TRIM5 gene from *Oryctolagus cuniculus* chromosome 1 [GenBank: NC_013669] (Forward 5’-CAAATTCATGAGCTGAAAAGGA-3’ and Reverse 5’AAGAGATGTACCCCAGGGTAAGAG-3’), and from *Ochotona princeps* unplaced genomic scaffold00040 [GenBank: NW_004535475] (Forward 5’-CAGAGGAAACCATTTGAAGCT-3’ and Reverse 5’-CTAGCAAAGCGTCATGGGT-3’). The primers designed according the rabbit TRIM5 were used in hare samples whereas the pika based primers were used in pika samples. The approximately 1.2Kb PCR product corresponds to all length of exon 6 and exon 7 (covering the entire PRYSPRY region).

Phusion® High-Fidelity DNA Polymerase (Finnzymes, Espoo, Finland) was used in the PCR amplification, the conditions included an initial denaturation (98ºC for 3min), 40 cycles of denaturation (98ºC for 30s), annealing (60ºC for 20s) and extension (72ºC for 45s) and a final extension (72ºC for 5min) for both hare and pika DNA. Amplicons sequencing was performed with the ABI PRISM BigDye Terminator v3.1 Cycle Sequencing Kit and according to manufacturer’s protocol; reactions were cleaned with Sephadex^™^ (GE Healthcare Life Sciences, UK) and applied on an ABI PRISM 310 Genetic Analyser (Life Technologies, Applied Biosystems, Carlsbad, CA, USA). PCR products were sequenced in both directions and, particularly for pika samples, an internal primer was also used (5’- ACTGGGAGGTGGATGTGTCT-3’).

Virtual transcripts of *Lepus* and *Ochotona* TRIM5 PRYSPRY domains were created by splicing together the exons reads (~ 600 bp) and have been deposited in the GenBank database under the following accession numbers: #MN605824 (*Ochotona hoffmanni*), #MN605825 (*Ochotona hyperborean)*, #MN605826 (*Ochotona mantchurica*), #MN605827 (*Ochotona pallasi*), #MN605828 (*Ochotona rutila*), #MN605829 (*Ochotona turuchanensis*), #MN605830 (*Ochotona alpina*), #MN605831 (*Ochotona pusilla*), #MN605832 (*Ochotona dauurica*), #MN605833 (*Lepus capensis*), #MN605834 (*Lepus castroviejoi*), #MN605835 (*Lepus corsicanus*), #MN605836 (*Lepus timidus*), #MN605837 (*Lepus townsendii*), #MN605838 (*Lepus americanus*), #MN605839 (*Lepus californicus*). *Lepus* and *Ochotona* samples have been used in previous publications [49–52]

### Sequence and Phylogenetic analyses

To complete the Lagomorpha dataset, besides the PCR-amplified *Lepus* and *Ochotona* PRYSPRY domain sequences, the TRIM5 nucleotide sequences of other leporid genera, including *Oryctolagus* and *Sylvilagus*, were obtained from NCBI database (http://www.ncbi.nlm.nih.gov). The PRYSPRY domain nucleotide sequences of Lagomorpha species were aligned in BioEdit Sequence Alignment Editor [53] using Clustal W [54], followed by manual corrections when necessary. Maximum likelihood (ML) phylogenetic reconstruction of Lagomorpha PRYSPRY domains was performed using PhyML v3.0 [27]. TIM3+G was identified as the best-fitting nucleotide substitution model, according to the Akaike information criterion (AIC) implemented in jModelTest v2.1.1 [55]. The support of the resulting nodes was estimated using 1000 bootstrap replicates.

### Molecular evolutionary analyses

The nucleotide sequences alignment for TRIM5 PRYSPRY domain was firstly tested for recombination, as this biological process can mislead molecular evolutionary analyses [56]. Coding sequences were scanned for recombination by using six methods (GENECONV, BootScan, MaxChi, Chimaera, SiScan and 3Seq) available in the RDP software, version 4.95 [57]. Nevertheless, no significant breakpoints were identified in the alignments.

To look for signatures of natural selection operating in the alignment, we used codeml of the PAML v4.9 package [58] and compared site-based models to determine if a model that allows positive selection (alternative model, M8) is a better fit to the data than a neutral model (null model, M7). The analysis was run with an initial ω ratio value of 1, and conducted with the F3×4 model of codon frequencies. Likelihood ratio test (LRT) was performed with two degrees of freedom to compare the fit of the two models by using the likelihood scores of the null neutral and alternative selection models. A Bayes empirical Bayes (BEB) approach was employed to detect codons with a posterior probability >95% of being under selection [59]. Five other methods, using HyPhy software implemented in the Datamonkey Web server [60, 61], were also applied to detect codons under selection: the Single Likelihood Ancestor Counting (SLAC) model, the Fixed Effect Likelihood (FEL) method, the Random Effect Likelihood (REL) method [62] and the recently described Mixed Effects Model of Evolution (MEME) [63] and Fast Unbiased Bayesian AppRoximation (FUBAR) [64] methods. To avoid a high false-positive rate [62], codons with p-values <0.1 for FEL and MEME models, Bayes Factor >50 for REL model and a posterior probability >0.90 for FUBAR were accepted as candidates for selection (S1 Appendix). For a more conservative approach, and as used previously [52, 65], only residues identified as being under positive selection in more than two ML methods were considered.

Sliding-window analysis for the Lagomorpha PRYSPRY domain was carried out using DnaSP v5.10 [66], for which a window length of 20 and a step size of 5 nucleotides were defined. The following non-synonymous substitutions per non-synonymous sites analyses were performed: *Sylvilagus bachmani* vs *Oryctolagus cuniculus*, *S*. *floridanus* vs *O*. *cuniculus* and *S*. *bachmani* vs *S*. *floridanus*; *O*. *dauurica* vs Ochotonidae family, *O*. *pusilla* vs Ochotonidae family and *O*. *dauurica* vs *O*. *pusilla*. A pairwise estimation of non-synonymous to synonymous substitution ratios (dN/dS) for the PRYSPRY v1 region of these same comparisons was performed on MEGA7 [33] using Nei-Gojobori (Jukes-Cantor correction) method.

### Primates PRYSPRY sequences

Using *Homo sapiens* TRIM5α PRYSPRY domain as template, we performed a BLAST search in GenBank (NCBI, http://BLAST.ncbi.nlm.nih.gov/) and Ensembl (http://www.ensembl.org/Multi/blastview) databases to obtain all the available Primate TRIM5α PRYSPRY sequences. The PRYSPRY sequences collected belong to Hominoidea, Cercopithecoidea and Platyrrhini groups. Sequences were aligned with Clustal W [54], implemented in BioEdit Sequence Alignment Editor [53], followed by visual inspection (S2 Appendix). The complete list of all Primate PRYSPRY sequences used in this study, together with accession numbers, is given in S3 Appendix.

## Supporting information

S1 Appendix Positive selection analyses for TRIM5 PRYSPRY domain of Lagomorphs. (DOCX)

S2 Appendix Amino acid alignment of the TRIM5 PRYSPRY domain of Hominoidea, Cercopithecoidea and Platyrrhini species. Amino acid sequences are grouped according to species genus. Variable loop “v1” from PRYSPRY domain is represented (grey box). Dots = identity with the PRYSPRY sequence from *Homo sapiens*.
(DOCX)

S3 Appendix List of the sequences of the TRIM5 PRYSPRY domain, available from NCBI and Ensemble databases and used in this study. (DOCX)
