# Supplementary material for: Not so unique to Primates: The independent adaptive evolution of TRIM5 in Lagomorpha lineage
Source: PLoS One. 2019 Dec 12;14(12):e0226202. doi: 10.1371/journal.pone.0226202 (PMC6907815; doi:10.1371/journal.pone.0226202)
Supplement: S1 Appendix — (DOCX) [file pone.0226202.s001.docx]

**S1 Table.** Positive selection analyses for TRIM5α PRYSPRY domain of Lagomorphs.

| Gene | Test of Selection | | | | Amino acids under Positive Selection | | | | | Total sites |
| --- | --- | --- | --- | --- | --- | --- | --- | --- | --- | --- |
|  | lnL ^a^ M7 | lnL^a^ M8 | 2Δ lnL^b^ | p-value | PAML^c^ | REL^d^ | FEL^e^ | MEME^e^ | FUBAR^f^ |  |
| TRIM5α | -2359.33 | -2332.78 | 26.55 | <0.001 | 26*,28*,32*,33**,35**,37**,38**,  39**,40*,41*,42**,44**,48**,49**,  50*,53**,54**,57**,58*,64*,90*,  92*,94*,97*,102*,103*,105*,  120**,123**,131*,134**,141*,  152*,176*,186*,195* | 26,32,33,35,36,38,  40,41,42,44,48,50,  53,54,57,58,102,109,  120,122,123,134,141,  163,176,186 | 38,42*,53*,  131,152 | 7,38,42,53*,  57**,99*,131,  152*,173*,186* | 35*,38*,41*,42*,44*,  48*,50*,53**,57,58*,  102*,120,123*,134,  141,176 | 13 |

a) lnL: log-likelihood scores.

b) 2ΔlnL: likelihood ratio test (LRT) to detect positive selection.

c) Codons with posterior probabilities >90% in the BEB analyses (*: P>95%; **: P>99%).

d) Codons with Bayes Factor >50.

e) Codons with significance level <0.1 (*: p<0.05; **: p<0.01).

f) Codons with posterior probabilities >0.90 (*: P>0.95; **: P>0.99).

**S1 Figure.** Nucleotide alignment used to infer positive selected sites in Lagomorpha species. Dots = identity with the PRYSPRY sequence of European rabbit.

10 20 30 40 50 60 70 80 90 100

....|....|....|....|....|....|....|....|....|....|....|....|....|....|....|....|....|....|....|....|

**European rabbit**  **GCCCAGCGCTATTGGGTTCACGTGACATTGACTCCAAGCAACAATCAAAATATTGTCGTTTCTGAGAATAAGAGACAAGTGATGTAT---GTGCATTACC**

**European rabbit (algirus)a1** **...........C...........................................................................---..........**

**European rabbit (algirus)a2** **...........C...........................................................................---..........**

**European hare a1**  **....G......C..................................................G........................---..........**

**European hare a2**  **....G......C..................................................G........................---..........**

**Iberian hare a1**  **....G......C..................................................G........................---..........**

**Iberian hare a2**  **....G......C..................................................G........................---..........**

**Brush rabbit**  **....G......C......G..T................................A................................---........TA**

**Eastern cottontail**  **....G......C......G..T.....................G......C....................................---..........**

**Snowshoe hare**  **....G......C..................................................G........................---..........**

**Black-tailed jackrabbit**  **....G......C..................................................G........................---..........**

**Cape hare**  **....G......C..................................................G........................---..........**

**Broom hare**  **....G......C..................................................G........................---..........**

**Corsican hare**  **....G......C..................................................G........................---..........**

**Mountain hare**  **....G......C..................................................G........................---..........**

**White-tailed jackrabbit**  **....G......C..................................................G........................---..........**

**American pika**  **.......A.C.C....CCA...........................CG.........A.................G....A.A...CACT.A.TT..T..**

**Hoffmann’s pika**  **.......A.C.C....CCA...........................CG.....C...A.................G....A.A...CACT.A.TT..T..**

**Northern pika**  **.......A.C.C....CCA...........................CG.........A.................G....A.A...CACT.A.TT..T..**

**Manchurian pika**  **.......A.C.C....CCA...........................CG.........A.................G....A.A...CACT.A.TT..T..**

**Palla’s pika**  **.......A.C.C....CCA...........................CG.........A.................G....A.A...CACT.A.TT..T..**

**Turkestan red pika**  **.......A.C.C....CCA...........................CG.....C...A.................G....A.A...CACT.A.TT..T..**

**Turuchan pika**  **.......A.C.C....CCA...........................CG........TA.................G....A.A...CACT.A.TT..T..**

**Alpine pika**  **.......A.C.C....CCA...........................CG.........A.................G....A.A...CACT.A.TT..T..**

**Steppe pika**  **.......ATC.C....C.A...........................C..........A.C...............A....A.AC..CATC.A.TTACT..**

**Daurian pika**  **.......A...C....C.............................C..........A.................AG.T.AGA...CATCAA.TTACC..**

110 120 130 140 150 160 170 180 190 200

....|....|....|....|....|....|....|....|....|....|....|....|....|....|....|....|....|....|....|....|

**European rabbit**  **ATCAACGTCTCAATTTATTTTCTCTTAGTGATGATCATGGCTTTCGATAT---GGGACTAGGCAGAATTATTTTGATGGCATCCTGGGTTGCCCAAGTAT**

**European rabbit (algirus)a1** **.G................................................---...............C...............................**

**European rabbit (algirus)a2** **.G................................................---...............C...............................**

**European hare a1**  **.GA...A.GG..G.C.T...CT.T...AG...A..T......G..A....GAG....T..A.......................................**

**European hare a2**  **.GA...A.GG..G.C.T...CT.G...AG...A..T......G..AG...GAG....T..A.......................................**

**Iberian hare a1**  **.GA...A.GG..G.C.T...CT.G...AG...A..T......G..AG...GAG....T..A.......................................**

**Iberian hare a2**  **.GA...A.GG..G...T...CT.G...AG...A..T......G..AG...GAG....T..A.......................................**

**Brush rabbit**  **.G....AA.CT.G...C...C..T...A....A..T...T.CG..A....GAG---...G........................................**

**Eastern cottontail**  **.G....A....GG...T------------------T....TCG..A....---........C..........A...........................**

**Snowshoe hare**  **.GA...A.GG..G...G...CT.G...AG...A..T......G..AG...GAG....T..A.......................................**

**Black-tailed jackrabbit**  **.GA...A.GG..G...G...CT.G...AG...A..T......G..AG...GAG....T..A.......................................**

**Cape hare**  **.GA...A.GG..G...T...CT.G...AG...A..T......G..AG...GAG....T..A............................C..........**

**Broom hare**  **.GA...A.GG..G...T...CT.G...AG...A..T......G..AG...GAG....T..A.......................................**

**Corsican hare**  **.GA...A.GG..G...T...CT.G...AG...A..T......G..AG...GAG....T..A.......................................**

**Mountain hare**  **.GA...A.GG..G...T...CT.G...AG...A..T......G..AG...GAG....T..A.......................................**

**White-tailed jackrabbit**  **.GA...A.GG..G...G...CT.G...AG...A..T......G..AG...GAG....T..A.......................................**

**American pika**  **..GG.AA.G.ATC...TGGC.T.AAA...ACAT..A..CTTACAG.GAT.CCA.TTCT..CAAG.CC...C.C...A......T....CCA....GC.T.**

**Hoffmann’s pika**  **..GG.AA.G.ATC...TGGC.T.AAA...ACAT..A..CTTACAG.GAT.CCA.TT.T..CAAG.CC.GTC.C...A......T....CCA....GC.T.**

**Northern pika**  **..GG.AA.G.ATC...TGGC.T.AAA...ACAT..A..CTTACAG.GAT.CCA.TT.T..CAAG.CC..TC.C...A......T....CCA....GC.T.**

**Manchurian pika**  **..GG.AA.G.ATC...TGGC.T.AAA...ACAT..A..CTTACAG.GAT.CCA.TT.T..CAAG.CC.GTC.C...A......T....CCA....GC.T.**

**Palla’s pika**  **..GG.AA.G.ATC...TGGC.T.AAA...ACAT..A..CTTACAG.GAT.CCA.TT.T..CAAG.CC..TC.C...A......T....CCA....GC.T.**

**Turkestan red pika**  **..GG.AA.G.ATC...TGGC.T.AAA...ACAT..A..CTTACAG.GAT.CCA.TT.T..CAAG.CC.GTC.C...A......T....CCA....GC.T.**

**Turuchan pika**  **..GG.AA.G.ATC...TGGC.T.AAA...ACAT..A..CTTACAG.GAT.CCA.TT.T..CAAG.CC..TC.C...A......T....CCA....GC.T.**

**Alpine pika**  **..GG.AA.G.ATC...TGGC.T.AAA...ACAT..A..CTTACAG.GAT.CCA.TT.T..CAAG.CC..GCCC...A......T....CCA....GC.T.**

**Steppe pika**  **..A..AA.GCACC...TAGC.T.ACA....CAT..AG.CCTACAGTTAT.CCA.TTTG..CAAG.CC...C.C...A......T....CCA....GC.T.**

**Daurian pika**  **..G..AA.AA.TC.---------GGA...ATGTT..TAATT.CA.CGCC.CCA.TTGT..CAAG.CC...CCC.A.A...........CCA....GC.T.**

210 220 230 240 250 260 270 280 290 300

....|....|....|....|....|....|....|....|....|....|....|....|....|....|....|....|....|....|....|....|

**European rabbit**  **CACATCAGGGAAACATTACTGGGAGGTAGATGTGTCTGGGAAAAGTGCCTGGATCCTAGGTGTATATGGCCCACCCTTATTGCAAACCACAACATCGTTT**

**European rabbit (algirus)a1** **....................................................................................................**

**European rabbit (algirus)a2** **....................................................................................................**

**European hare a1**  **.............................................................................C...........T..A.......**

**European hare a2**  **.............................................................................C...........T..A.......**

**Iberian hare a1**  **.............................................................................C...........T..A.......**

**Iberian hare a2**  **.............................................................................C..............A.......**

**Brush rabbit**  **................................................................CG.....T............................**

**Eastern cottontail**  **................................................................CG.....T..................G.....A...**

**Snowshoe hare**  **.............................................................................C..............A.......**

**Black-tailed jackrabbit**  **.............................................................................C..............A.......**

**Cape hare**  **.............................................................................C..............A..TC...**

**Broom hare**  **.............................................................................C..............A.......**

**Corsican hare**  **.............................................................................C..............A.......**

**Mountain hare**  **.............................................................................C..............A.......**

**White-tailed jackrabbit**  **.............................................................................C..............A.......**

**American pika**  **.............A.............G..........A.....C.........T..C..G..C.G.CAA...AAG...GGC.C...ACA....C....C**

**Hoffmann’s pika**  **.............A.............G..........A.....C.........T..C..G..C.G.CAA...AAG...GGC.C...ACA....C....C**

**Northern pika**  **.............A.............G..........A.....C.........T..T..G..C.G.CAA...AAG...GGC.C...ACA....C....C**

**Manchurian pika**  **.............A.............G..........A.....C.........T..C..G..C.G.CAA...AAG...GGC.C...ACA....C....C**

**Palla’s pika**  **.............A.............G..........A.....C.........T..T..G..C.G.CAA...AAG...GGC.C...ACA....C....C**

**Turkestan red pika**  **.............A.............G..........A.....C.........T..C..G..C.G.CAA...AAG...GGC.C...ACA....C....C**

**Turuchan pika**  **.............A.............G..........A.....C.........T..T..G..C.G.CAA...AAG...GGC.C...ACA....C....C**

**Alpine pika**  **.............A.............G..........A.....C.........T..T..G..C.G.CAA...AAG...GGC.C...ACA....C....C**

**Steppe pika**  **.............A.............G..........A.....C.........T..C..G..C.G.CAA...AAG...GGC.C...ACA....C....C**

**Daurian pika**  **.............A........................A.....C........CT..C..G..C.G.CAA...AAG...GGC.C...ACA...GC..C.C**

310 320 330 340 350 360 370 380 390 400

....|....|....|....|....|....|....|....|....|....|....|....|....|....|....|....|....|....|....|....|

**European rabbit**  **GCTTATGAACAAGTTTCAAAGTATCGGCCTATAAATGGCTACTGG---GTAATAGGGTTGCAAATTCAATATATTTCTTTTGAGGAAAATGCCATTTCCT**

**European rabbit (algirus)a1** **.............................................---....................................................**

**European rabbit (algirus)a2** **.............................................---....................................................**

**European hare a1**  **.......G....T................................---..........G.....A..............A..........T......G..**

**European hare a2**  **.......G....T............A...................---..........G.....A..............A..........T......G..**

**Iberian hare a1**  **.......G....T............A...................---..........G.....A..............A..........T......G..**

**Iberian hare a2**  **.......G....T............A...................---..........G.....A..............A..........T.........**

**Brush rabbit**  **....T..G.....C...............................---................A.A.................................**

**Eastern cottontail**  **....T..G.....................................---................A.G.................................**

**Snowshoe hare**  **.......G....T............A...................---..........G.....A..............A..........TG........**

**Black-tailed jackrabbit**  **.......G....T............A...................---..........G.....A..............A..........TG........**

**Cape hare**  **.......G....T............A...................---..........G.....A..............A..........T.........**

**Broom hare**  **.......G....T............A...................---..........G.....A..............A..........T.........**

**Corsican hare**  **.......G....T............A...................---..........G.....A..............A..........T.........**

**Mountain hare**  **.......G....T............A...................---..........G.....A..............A..........T.........**

**White-tailed jackrabbit**  **.......G....T............A...................---..........G.....A..............A..........T.........**

**American pika**  **TT.CC.C......G......A..CAAA...G..C...........GTTA..GG.TTACA.GGTCAGTCTC..G...T..AC...A.....C......T.G**

**Hoffmann’s pika**  **TT.CC.C......G......A..CAAA...G..C...........GTCA..GG.TTACA.GGTCAGTCTC..G...T..AC.........C......T.A**

**Northern pika**  **TT.CC.C......G......A..CAAA...G..C...........GTCA..GG.TTACA.GGTCAGTCTC..G...T..AC.........C......T.A**

**Manchurian pika**  **TT.CC.C......G......A..CAAA...G..C...........GTCA..GG.TTACA.GGTCAGTCTC..G...T..AC.........C......T.A**

**Palla’s pika**  **TT.CC.C......G......A..CAAA...G..C...........GTCA..GG.TTACA.GGTCAGTCTC..G...T..AC.........C......T.A**

**Turkestan red pika**  **TT.CC.C......G......A..CAAA...G..C...........GTCA..GG.TTACA.GGTCAGTCTC..G...T..AC.........C......T.A**

**Turuchan pika**  **TT.CC.C......G......A..CAAA...G..C...........GTCA..GG.TTACA.GGTCAGTCTC..G...T..AC.........C......T.A**

**Alpine pika**  **TT.CC.C......G......A..CAAA...G..C...........GTCA..GG.TTACA.GGTCAGTCTC..G...T..AC.........C......T.A**

**Steppe pika**  **T..CT.C......G......A..CAAA...GC.C...........GTCA..GG.TTACA.GGTCCATCTC..G...T..A........CGC......T.A**

**Daurian pika**  **TT.CC.C......G.....TA..CAAA...G..CG..........GTCA..GG.TTACG.GGTCAGTCTC..G...T..AC........AC......T.G**

410 420 430 440 450 460 470 480 490 500

....|....|....|....|....|....|....|....|....|....|....|....|....|....|....|....|....|....|....|....|

**European rabbit**  **TAACACCCATTGTTCCTCCTTCTCGTATTGGAGTTTTCATAGACTATGAGGCTGGCATTGTCTCATTTTTCAGTGTTACACAGCACAAGTTTCTCATATA**

**European rabbit (algirus)a1** **....................................................................................................**

**European rabbit (algirus)a2** **....................................................................................................**

**European hare a1**  **.......................T..........................................................A....C.........C..**

**European hare a2**  **.......................T..........................................................A....C.........C..**

**Iberian hare a1**  **.......................T..........................................................A....C.........C..**

**Iberian hare a2**  **.......................T..........................................................A....C.........C..**

**Brush rabbit**  **..................T..................................A............................A..............C..**

**Eastern cottontail**  **...A..................................................C...........................A..............C..**

**Snowshoe hare**  **.......................T..........................................................A....C.........C..**

**Black-tailed jackrabbit**  **.......................T..........................................................A....C.........C..**

**Cape hare**  **.......................T..........................................................A....C..G......C..**

**Broom hare**  **.......................T..........................................................A....C.........C..**

**Corsican hare**  **.......................T..........................................................A....C.........C..**

**Mountain hare**  **.......................T..........................................................A....C.........C..**

**White-tailed jackrabbit**  **.......................T..........................................................A....C.........C..**

**American pika**  **.G..CTTG.CAA.G...T..CA...C.....G......C.C................CC.....C.................C.CAGTA.A......G..**

**Hoffmann’s pika**  **.G..CTTG.CAA.....T..CG...C.....G......C.C................CC.....C..C..............C.CAGTA.A......G..**

**Northern pika**  **.G..CTTG.CAA.....T..CG...C.....G......C.C................CC.....C..C..............C.CAGTA.A......G..**

**Manchurian pika**  **.G..CTTG.CAA.....T..CG...C.....G......C.C................CC.....C..C..............C.CAGTA.A......G..**

**Palla’s pika**  **.G..CTTG.CAA.....T..CG...C.....G......C.C................CC.....C..C..............C.CAGTA.A......G..**

**Turkestan red pika**  **.G..CTTG.CAA.....T..CG...C.....G......C.C................CC.....C..C..............C.CAGTA.A......G..**

**Turuchan pika**  **.G..CTTG.CAA.....T..CG...C.....G......C.C................CC.....C..C..............C.CAGTA.A......G..**

**Alpine pika**  **.G..CTTG.CAA.....T..CG...C.....G......C.C................CC.....C..C..............C.CAGTA.A......G..**

**Steppe pika**  **.G..CTTG.CAA.....T..CG...C.....G......C.CA...........A...C.A....C.................C.CA.TA.A......C..**

**Daurian pika**  **AG..CTTG.CAAC....T.C.G...C.....G......C.C................C......C.............T...C.CA.TA.A......C..**

510 520 530 540 550 560 570 580 590

....|....|....|....|....|....|....|....|....|....|....|....|....|....|....|....|....|....|....

**European rabbit**  **TAAGTTCTCTGCGTGTTCTTTTTCTAAGGAAGTTTTTCCATATTTCAATCCTATGCATTGTCCAAAGCCCATGACAATCTGCGAGTTGAGCTGT**

**European rabbit (algirus)a1** **..............................................................................................**

**European rabbit (algirus)a2** **..............................................................................................**

**European hare a1**  **............A.........G..G...............................A........................C..A........**

**European hare a2**  **............A.........G..G...............................A........................C..A........**

**Iberian hare a1**  **............A.........G..G...............................A........................C..A........**

**Iberian hare a2**  **............A.........G..G...............................A........................C..A........**

**Brush rabbit**  **.......G....A............................................A........................C...........**

**Eastern cottontail**  **......TG....A............................................A........A........G..................**

**Snowshoe hare**  **............A.........G..G...............................A........................C..A........**

**Black-tailed jackrabbit**  **............A.........G..G...............................A........................C..A........**

**Cape hare**  **............A.........G..G...............................A........................C..A........**

**Broom hare**  **............A.........G..G...............................A........................C..A........**

**Corsican hare**  **............A.........G..G...............................A........................C..A........**

**Mountain hare**  **............A.........G..G...............................A........................C..A........**

**White-tailed jackrabbit**  **............A.........G..G...............................A........................C..A........**

**American pika**  **...A.......GA..............T...........................ACG.......GA........GC.T...------------**

**Hoffmann’s pika**  **...A.......GA..............T...........................ACG.......GA........GC.T...------------**

**Northern pika**  **...A.......GA..............T...........................ACG.......GA........GC.T...------------**

**Manchurian pika**  **...A.......GA..............T...........................ACG.......GA........GC.T...------------**

**Palla’s pika**  **...A.......GA..............T...........................ACG.......GA........GC.T...------------**

**Turkestan red pika**  **...A.......GA..............T...........................ACG.......GA........GC.T...------------**

**Turuchan pika**  **...A.......GA..............T...........................ACG.......GA........GC.T...------------**

**Alpine pika**  **...A.......GA..............T...........................ACG.......GA........GC.T...------------**

**Steppe pika**  **...........GA.........C...GT.............................G.......GA........GC.T...------------**

**Daurian pika**  **..GA.......G....AT.......G.TA......A.....................G....A..GA........GC.T...------------**
