# Supplementary material for: Not so unique to Primates: The independent adaptive evolution of TRIM5 in Lagomorpha lineage
Source: PLoS One. 2019 Dec 12;14(12):e0226202. doi: 10.1371/journal.pone.0226202 (PMC6907815; doi:10.1371/journal.pone.0226202)
Supplement: S2 Appendix — Amino acid sequences are grouped according to species genus. Variable loop “v1” from PRYSPRY domain is represented (grey box). Dots = identity with the PRYSPRY sequence from Homo sapiens. (DOCX) [file pone.0226202.s002.docx]

**S2 Appendix.** Amino acid alignment of the TRIM5α PRYSPRY domain of Hominoidea, Cercopithecoidea and Platyrrhini species. Amino acid sequences are grouped according to species genus. Variable loop “v1” from PRYSPRY domain is represented (grey box). Dots = identity with the PRYSPRY sequence from *Homo sapiens*.

**Hominoidea group**

10 20 30 40 50 60 70 80 90 100 110 120 130

....|....|....|....|....|....|....|....|....|....|....|....|....|....|....|....|....|....|....|....|....|....|....|....|....|....|....|....

**Homo sapiens**  **PDLKGMLEVFRELTDVRRYWVDVTVAPNNISCAVISEDKRQVSSPKPQIIYGARGTRYQTFVNFNYCTGILGSQSITSGKHYWEVDVSKKTAWILGVCAGFQPDAMCNIEKNENYQPKYGYWVIGLEEGVKCSAFQDSS**

**Pan troglodytes**  **......................................M......................M............................S..............................................G.**

**Pan paniscus**  **......................................M......................M............................S..............................................G.**

**Pongo abelii**  **.N..........................D..Y......M....C.E.......Q..T...Y.............................S...............Y...Q......Q...................G.**

**Pongo pygmaeus**  **.N..........................D..Y......M....C.E.......Q..T...Y...S.........................S...............Y...Q......Q...................G.**

**Hylobates agilis**  **--------------------...........Y......M......E.....E.Q..IS................................S.........L.....Y...Q.....................N....G.**

**Hylobates pileatus**  **--------------------...........Y......M......E.....E.Q..IS................................S.........L.....Y...Q.....................N....G.**

**Hylobates lar**  **A..QVT...L...R.................Y......M......E.....E.Q..IS................................S...............Y...Q.....................N....G.**

**Nomascus leucogenys** **A...V...EL...R..QH.............Y......M......E.....E.Q..IS......S.........................S.........L.....Y...Q.................R...N....G.**

**Nomascus gabriellae** **--------------------...........Y......M......E.....E.Q..IS......S.........................S.........L.....Y...Q.................R...N....G.**

140 150 160 170 180 190 200 210

|....|....|....|....|....|....|....|....|....|....|....|....|....|....|....|

**Homo sapiens**  **FHTPSVPFIVPLSVIICPDRVGVFLDYEACTVSFFNITNHGFLIYKFSHCSFSQPVFPYLNPRKCGVPMTLCSPSS**

**Pan troglodytes**  **.....A...................................S..................................**

**Pan paniscus**  **.....A......................................................................**

**Pongo abelii**  **..N..A...........................................................R..........**

**Pongo pygmaeus**  **..N..A...........................................................R..........**

**Hylobates agilis**  **S....A........K..........................V.......................T..........**

**Hylobates pileatus**  **S....A........K..........................V.......................T..........**

**Hylobates lar**  **S....A........K..................................................T..........**

**Nomascus leucogenys** **.....A........N................L.........V.......................T..........**

**Nomascus gabriellae** **.....A........N................L.........V.......................T..........**

**Cercopithecoidea (Old World Monkeys)**

10 20 30 40 50 60 70 80 90 100 110 120 130

....|....|....|....|....|....|....|....|....|....|....|....|....|....|....|....|....|....|....|....|....|....|....|....|....|....|....|....

**Homo sapiens**  **PDLKGMLEVFRELTDVRRYWVDVTVAPNNISCAVISEDKRQVSSPKPQIIYGARGTRYQ--TFVNFNYCTGILGSQSITSGK--------------------HYWEVDVSKKTAWILGVCAGFQPDAMCNIEKNENYQP**

**Rhinopithecus roxellana** **.......DM...............L......H...A.........N...M.Q.P..LF.--SLK..I....V..........--------------------..........S...............Y...Q......**

**Rhinopithecus bieti**  **.......DM...............L......H...A.........N...M.Q.P..LF.--SIK..I....V..........--------------------..........S...............Y...Q......**

**Cercopithecus ascanius**  **.......DM...............L......H...A........RNS..M.Q.P..LFG--SLT.......V..........--------------------..........S..............TY...Q......**

**Cercopithecus cephus**  **.......DM...............L......H...A........RNS..M.Q.P..LFG--SLT.......V..........--------------------..........S..............TY...Q......**

**Cercopithecus wolfi**  **.......DM...............L......H...A........RN...M.R.P.KLF.--SRT.......V..........--------------------..........S..............TY...Q......**

**Cercopithecus neglectus** **.......DM......A.C......L....F.H...A........RNS..M.Q.P..LFG--SLP.......V..........--------------------..........S...............Y...QK.....**

**Chlorocebus aethiops**  **.......DM...............L......H...A.......YQN...M.Q.P.SSFG--SLT.......V........R.LTNFNYCTGVLGSQSITSGK..........S..............TY...Q......**

**Chlorocebus pygerythrus** **--.....DM...............L......H...A.......YRN...M.QSP.SLFG--SLT..S....VP.........LTNFNYCTGVLGSQSITSGK..........S..............TY...Q......**

**Chlorocebus sabaeus**  **--------------------....L......H...A.......YQN...M.Q.P.SLFG--SLT.......V........R.LTNFNYCTGVLGSQSITSGK..........S..............TY...Q......**

**Chlorocebus tantalus**  **.......DM...............L......H...A.......YQN...M.Q.P.SSFG--SLT.......V........R.LTNFNYCTGVLGSQSITSGK..........S..............TY...Q......**

**Cercocebus atys**  **.......DM...............L......H...A........RN...M.Q....LFSFPSHT.......V..........--------------------..........S...............Y...Q......**

**Cercocebus torquatus**  **.......DM...............L......H...A........RN...M.Q.Q..LFSFPSHT.......V..........--------------------..........S...............Y...Q......**

**Papio anubis**  **.......DM...............L......H...A........RN...T.Q.P..LFSFPSLT.......V..........--------------------..........S...............Y...Q......**

**Papio cynocephalus**  **--------------------....L......H...A........RN...T.Q.P..LFSFPSLT.......V...P......--------------------..........S...............Y...Q......**

**Papio hamadryas**  **------------............L......H...A........RN...T.Q.P..LFSFPSLT.......V...P......--------------------..........S...............Y...Q......**

**Macaca assamensis**  **--.....DM......A........L......H...A........RN...M.Q.P..LFTFPSLT.......V..........--------------------..........N...........S...Y...Q......**

**Macaca fascicularis**  **--.....DM......A........L......H...A........RN...V.QSP..LF.--SLT.......V..........--------------------..........S...........S.......Q......**

**Macaca mulatta**  **.......DM......A........L......H...A........RN...M.Q.P..LFTFPSLT.......V..........--------------------..........S...........S...Y...Q......**

**Macaca nigra**  **------------...A........L......HV..A........RN...M.Q.P..LF.--SLT..S....V..........--------------------..........S...........S...Y...Q......**

**Macaca sylvanus**  **.......DM......A.C......L....M.H...A........RN...M.QTP..LFTFPSLT.......V.....L....--------------------..........S...........L...Y...Q......**

**Macaca thibetana**  **--.....DM......A........L......H...A........RN...M.Q.P..LF.--SLT.......V..........--------------------..........S...........S...Y...Q......**

140 150 160 170 180 190 200 210 220 230

|....|....|....|....|....|....|....|....|....|....|....|....|....|....|....|....|....|....|....|..

**Homo sapiens**  **KYGYWVIGLEEGVKCSAFQDSSFHTPSVPFIVPLSVIICPDRVGVFLDYEACTVSFFNITNHGFLIYKFSHCSFSQPVFPYLNPRKCGVPMTLCSPSS**

**Rhinopithecus roxellana** **.........QK...Y.V...G.S...FA..................V.......................Q....K...........T..........**

**Rhinopithecus bieti**  **.........QK...Y.V...G.S...FA..................V.......................Q....K...........T..........**

**Cercopithecus ascanius**  **.........Q....Y.....G.SY.TFA..................V.......................Q....K.I.........T..........**

**Cercopithecus cephus**  **.........Q....Y.....G.SY.TFA..................V.......................Q....K.I.........T..........**

**Cercopithecus wolfi**  **.........Q....Y.....G.SY.TFA.L................V.......................Q....E...........T..........**

**Cercopithecus neglectus** **.........QD...Y.....G.SF.SFA..................V.......................Q....K.I.........T..........**

**Chlorocebus aethiops**  **.........Q..D.Y.V.....S...FA..................V.......................Q....K...........T..........**

**Chlorocebus pygerythrus** **.........Q..D.Y.V...G.S...FA..................V.......................Q....K...........T..........**

**Chlorocebus sabaeus**  **.........Q..D.Y.V.....S...FA..................V.......................Q....K...........T..........**

**Chlorocebus tantalus**  **.........Q..D.Y.V...G.S...FA..................V.......................Q....K...........T..........**

**Cercocebus atys**  **.........Q....Y.V...G.S...FA...A..............V.......................Q....K...........T..........**

**Cercocebus torquatus**  **.........Q....Y.V...G.S...FA..................V.......................Q....K...........T..........**

**Papio anubis**  **.........Q....Y.V...G.S...FA..................V.......................Q....K...........T..........**

**Papio cynocephalus**  **.........QK...Y.V...G.S...FA..................V.......................Q....K...........T..........**

**Papio hamadryas**  **.........Q....Y.V...G.S...FA..................V.............S.........Q....K...........T..........**

**Macaca assamensis**  **.........Q....Y.V...G.S...FA..................V.......................Q....K...........T..........**

**Macaca fascicularis**  **.........Q....Y.V...G.L...FA..................V.......................Q....K...........T..........**

**Macaca mulatta**  **.........Q....Y.V...G.S...FA..................V.......................Q....K...........T..........**

**Macaca nigra**  **.........Q....Y.V...G.S...FA..................V.......................Q....K...........T..........**

**Macaca sylvanus**  **.........Q....Y.V...G.S...FA..................V.......................Q....K...........T..........**

**Macaca thibetana**  **.........Q....Y.V...G.S...FA..................V.......................Q....K...........T..........**

**Platyrrhini group**

10 20 30 40 50 60 70 80 90 100 110 120 130

....|....|....|....|....|....|....|....|....|....|....|....|....|....|....|....|....|....|....|....|....|....|....|....|....|....|....|....

**Homo sapiens**  **PDLKGMLEVFRELTDVRRYWVDVTVAPNNISCAVISEDKRQVSSPKPQIIYGARGTRYQTFVNFNYCTGILGSQSITSGKHYWEVDVSKKTAWILGVCAGFQPDAMCNIEKNENYQPKYGYWVIGLEEGVKCSAFQD--**

**Alouatta belzebul**  **----------------------..LI..HP..T.........RYQEQIHH---------LSMEVK.FY.....P..............N.S.......VSLKC--IG.FPGI......N.......QDADNY.....AV**

**Alouatta sara**  **.......Q..K..KE.QC..AH..LI..HP..T.......E.RYQEQIHHH--------PSMEVK.FY.....P..............N.S.......VSLKC--IG.FPGI.....QN.......RNADNY.....AV**

**Aotus azarae**  **---------------------H..LV.SHP..T.....E...RYQERIYQ---------P..KVK.FC.V...P..............N.SE......VSLKRT.S.SVPRI......N.S.....WNADNY.....AA**

**Aotus trivirgatus**  **---------------------------SHP..T.....E...RYQ.RIYQ---------P.LKVK.FC.V...P..............N.SE......VSLKRT.S.SVPRI..D...N.......RNADNY.....AV**

**Saimiri sciureus**  **....R..Q.LK...E.Q...AH..LV.SHP.YTI....G...RYQ..IRH---------LL.KVQ.FY.V...P..............N.R..T....VSLKCT.NQSVSGT......N.......RNAGNYR...SSF**

**Saimiri boliviensis boliviensi** **....R..Q.LK...E.Q...AH..LV.SHP.YTI....G...RYQ..IRH---------LL.KVQ.FY.V...P..............N.R..T....VSLKCT.NQSVSGT......N.......RNAGNYR...SSF**

**Saimiri ustus**  **---------------------H..LV.SHP.YTI....G...RYQ..IRH---------LL.KVQ.FY.V...P..............N.R..T....VSLKCT.NQSVSGT......N.......RNAGNYR...SSF**

**Callithrix jacchus**  **.......QA.K...E.Q...AH..LV.SHP..T.....E...RYQV.IHQ---------PL.KVK.FY.V...L..............N.RG......GSLKCN.KW.VLRP......N.......RNTDNY.....AV**

**Callithrix kuhlii**  **---------------------H..LV.SHP..T.....E...RYQV.IHQ---------PL.KVK.FY.V...L..............N.RG......GSLKCN.KW.VLRP......N.......RNTDNY.....AV**

**Callithrix penicillata**  **---------------------H..LV.SHP..T.....E...RYQV.IHQ---------PL.KVK.FY.V...L..............N.RG......GSLKCN.KW.VLRP......N.......RNTDNY.....AV**

**Mico argentatus**  **---------------------H..LV.SHP..T.....E...RYQV.IHQ---------PL.KVK.FY.V...L..............N.RG......GSLKCN.KW.VLRP......N.......RNTDNY.....AV**

**Mico emiliae**  **---------------------H..LV.SHP..T.....E...RYQV.IHQ---------PL.KVK.FY.V...L..............N.RG......GSLKCN.KW.VLRP......N.......RNTDNY.....AV**

**Mico humeralifer**  **---------------------H..LV.SHP..T.....E...RYQV.IHQ---------PL.KVK.FY.V...L..............N.RG......GSLKCN.KW.VLRP......N.......RNTDNY.....AV**

**Leontopithecus chrysopygus**  **---------------------H..LV.SHP.Y.....N....RYQA.IHQ---------PL.KVK.FY.V...P..............N.RD......VSLKCNEKW.VLRP......N.......RYADNYC....AV**

**Leontopithecus rosalia**  **---------------------H..LV.SHP.Y..........RYQA.IHQ---------PL.KVK.FY.V...P..............N.RD......VSLKCNEKW.VLRP......N.......RYADNYC....AI**

**Saguinus bicolor**  **---------------------H..LV.SHP.Y......G...RYQFQIHQ---------PS.KV..FY.V...P..............N.R.......VSLKCN.KW.VLRP......N.......QNTNNY.....AV**

**Saguinus imperator**  **---------------------H..LV.SHP.Y......E...RYQFQIHQ---------PS.KV..FY.V...P.............TN.RD....I.VS.KCN.KW.VLRP......N.......QNTNNY.....AV**

**Saguinus labiatus**  **....A..QA.K...E.Q...AH..LV.SHP.Y......E...RYQFQIHQ---------PS.KV..FY.V...P.............TN.RD....I.VS.KCN.KW.VLRP......N.......QNTNNY.....AV**

**Saguinus mystax**  **---------------------H..LV.SHP.Y......E...RYQFQIHQ---------PS.KV..FY.V...P.............TN.RD..R.I.VT.KCN.KW.VL.P......N..G....RNTNNY.....AV**

**Saguinus oedipus**  **.......QA.K...E.Q...AH..LV.SHP.YT.....E...RYQF.IHQ---------PS.KV..FY.V...P..............N.R.......VSLKYN.KW.VLRP......N.......QNTNNY.....AV**

**Pithecia irrorata**  **---------------------H..LV.SHP........E...RYQERIHQ---------S.GKVK.FY.V...P..R...........N.S.......VSLKCT.NR.GPRM......N.......WNAGNY.....SV**

**Pithecia pithecia**  **.......Q..K...E.Q....H..LV.SHL........E...RYQERIHQ---------S.GKVK.FY.V...P..R...........N.S.......VSLKCT.NR.GPRI......N.......WNAGNY.....SV**

**Callicebus donacophilus**  **.......Q.SK...E.Q...AH..LVASHP.R......E...RYQEWIHQ---------SSGRVK.FY.V...P..............N.S.......VSLKCA.NR.GPGV......N.......RNADNY.....SV**

**Callicebus moloch**  **.......Q.SK...E.Q...AH..LVASHP.R......E...RYQEWIHQ---------SSGRVK.FY.V...P..............N.S.......VSLKCA.NR.GPGV......N.......RNADNY.....SV**

140 150 160 170 180 190 200 210 220 230 240 250 260 270 280

|....|....|....|....|....|....|....|....|....|....|....|....|....|....|....|....|....|....|....|....|....|....|....|....|....|....|....|....|....|.

**Homo sapiens**  **-------SSFHTP--------------------------------------------------------------SVPFIVPLSVIICPDRVGVFLDYEACTVSFFNITNHGFLIYKFSHCSFSQPVFPYLNPRKCGVPMTLCSPSS**

**Alouatta belzebul**  **PETENYQPKTRNRFTGLQNADNYSAFQNAFPGIQSYQPKKSHLFTGLQNLSNYNAFQNKVQYNYSDFQDDSLSTP.A.L....FMT...K.................V.SN.Y......N.Q..Y.....FS.MT.EL....-----**

**Alouatta sara**  **PETENYQPKNRNRFTGLQNADNCSAFQNAFPGIQSYQPKKSHLFTGLQNLSNYNAFQNKVQYNYIDFQDDSLSTP.A.L....FMT...K.................V.SN.Y......N.Q..Y.....FS.MT.EL.........**

**Aotus azarae**  **EYSDFQDG.CS..--------------------------------------------------------------.A.L....FMT...N.................V..N........N.H.CY.....FS.MT.E----------**

**Aotus trivirgatus**  **EYSDFQDG.RS..--------------------------------------------------------------.A.L....FMT...N.................V..N........N.H.CY.....FS.MT.EL.........**

**Saimiri sciureus**  **EFRDFLAG.RL.L--------------------------------------------------------------.P.L....FMT...N..........R.I....V.SN........D.H..Y.....F..MT.EL.......R.**

**Saimiri boliviensis boliviensi** **EFRDFLAG.RL.L--------------------------------------------------------------.P.L....FMT...N..........R.I....V.SN........D.H..Y.....F..MT.EL.........**

**Saimiri ustus**  **EFRDFLAG.RL.L--------------------------------------------------------------.P.L....FMT...N..........R.I....V.SN........D.H..Y.....F..MT.EL.......R.**

**Callithrix jacchus**  **KYSDFQDG.RSIS--------------------------------------------------------------.G.L....FMT...N.................V.SN........N.H..Y.....FS.MT.EL.........**

**Callithrix kuhlii**  **KYSDFQDG.RSIS--------------------------------------------------------------.G.L....FMT...N.................V.SN........N.P..Y.....FS.MT.EL.........**

**Callithrix penicillata**  **KYSDFQDG.RSIS--------------------------------------------------------------.G.L....FMT...N.................V.SN........N.H..Y.....FS.MT.EL.........**

**Mico argentatus**  **KYSDFQDG.RSIS--------------------------------------------------------------.G.L....FMT...N.................V.SN........N.H..Y.....FS.TT.EL.........**

**Mico emiliae**  **KYSDFQDG.RSIS--------------------------------------------------------------.G.L....FMT...N.................V.SN........N.H..Y.....FS.TT.EL.........**

**Mico humeralifer**  **KYSDFQDG.RSIS--------------------------------------------------------------.G.L....FMT...N.................V.SN........N.H..Y.....FS.TT.EL.........**

**Leontopithecus chrysopygus**  **KYSDFQD..RS..--------------------------------------------------------------.A.LV...FMT.F.N.......C........DV..N........N.H..Y.....FS.MT.EL.........**

**Leontopithecus rosalia**  **KYSDFQD..RS..--------------------------------------------------------------.A.LV...FMT.F.N.......C........DV..N........N.H..Y.....FS.MT.EL.........**

**Saguinus bicolor**  **KYSDFQIG.RS.A--------------------------------------------------------------...L....FMT...N.................V..N........N.H..Y.....FS.VT.EL.........**

**Saguinus imperator**  **KYSDFQIG.RS.A--------------------------------------------------------------...L....FMT.Y.N.................V..N........N.H..Y.....FS.MT.EL.........**

**Saguinus labiatus**  **KYSDFQIG.RS.A--------------------------------------------------------------...L....FMT.Y.N.................V..N........N.H..Y.....FS.MT.EL.........**

**Saguinus mystax**  **KYSDFQIG.RC.A--------------------------------------------------------------...L....FMT.Y.N.................V.SN........N.H..Y..I..FS.TT.EL.........**

**Saguinus oedipus**  **KYSDFQIG.RS.A--------------------------------------------------------------...L....FMT...N.................V..N........N.H..Y.....FS.VT.EL.........**

**Pithecia irrorata**  **KYSDFQDG.HS.T--------------------------------------------------------------YG.L....FMT...N.................V.SN........N.R..DS....FS.MT.EL.......R.**

**Pithecia pithecia**  **KYSDFQDG.HSAT--------------------------------------------------------------YG.L....FMT...N.................V.SN........N.R..DS....FS.MT.EL.......R.**

**Callicebus donacophilus**  **KYNDFQDG.RS.T--------------------------------------------------------------YA.L....FMT...N.................V.SN........N.H..Y.....FS.MT.EL.......R.**

**Callicebus moloch**  **KYNDFQDG.RS.T--------------------------------------------------------------YA.L....FMT...N.................V.SN........N.H..Y.....FS.MT.EL.......R.**
