# Supplementary material for: Not so unique to Primates: The independent adaptive evolution of TRIM5 in Lagomorpha lineage
Source: PLoS One. 2019 Dec 12;14(12):e0226202. doi: 10.1371/journal.pone.0226202 (PMC6907815; doi:10.1371/journal.pone.0226202)
Supplement: S3 Appendix — (DOCX) [file pone.0226202.s003.docx]

**S3 Appendix.** List of the sequences of the TRIM5α PRYSPRY domain, available from NCBI and Ensemble databases and used in this study.

| Species name | Common name | Accession no. |
| --- | --- | --- |
| *Homo sapiens* | Human | NP_149023.2 |
| *Pan troglodytes* | Chimpanzee | NP_001012668.1 |
| *Pan paniscus* | Pygmy chimpanzee | ABC33738.1 |
| *Pongo abelii* | Sumatran orangutan | XP_024110124.1 |
| *Pongo pygmaeus* | Bornean orangutan | AAY23161.2 |
| *Hylobates agilis* | Agile gibbon | AJO15916.1 |
| *Hylobates pileatus* | Pileated gibbon | AJO15914.1 |
| *Hylobates lar* | Lar gibbon | AAY23162.1 |
| *Nomascus leucogenys* | Northern white-cheeked gibbon | AAX86678.1 |
| *Nomascus gabriellae* | Yellow-cheeked gibbon | AJO15915.1 |
| *Rhinopithecus roxellana* | Golden snub-nosed monkey | XP_010362850.1 |
| *Rhinopithecus bieti* | black snub-nosed monkey | XP_017742798.1 |
| *Cercopithecus ascanius* | Red-tailed monkey | ALB36909.1 |
| *Cercopithecus cephus* | Moustached monkey | ALB36907.1 |
| *Cercopithecus wolfi* | Wolf's mona monkey | ALB36905.1 |
| *Cercopithecus neglectus* | De Brazza's monkey | ALB36910.1 |
| *Chlorocebus aethiops* | Grivet monkey | AAT48103.1 |
| *Chlorocebus pygerythrus* | Vervet monkey | AY740612.1 |
| *Chlorocebus tantalus* | Tantalus monkey | AAT10388.2 |
| *Chlorocebus sabaeus* | Green monkey | XM_008019878.1 |
| *Cercocebus atys* | Sooty mangabey | ABL14047.1 |
| *Cercocebus torquatus* | collared mangabey | ALB36906.1 |
| *Papio Anubis* | olive baboon | NP_001106102.1 |
| *Papio cynocephalus* | Yellow baboon | AY710295.1 |
| *Papio hamadryas* | Hamadryas baboon | HM468444.1 |
| *Macaca assamensis* | Assam macaque | AH014832.2 |
| *Macaca fascicularis* | Crab-eating macaque | NM_001283295.1 |
| *Macaca mulatta* | Rhesus macaque | AAX86682.1 |
| *Macaca sylvanus* | Barbary macaque | ADQ48011.1 |
| *Macaca nigra* | Celebes black macaque | HM468436.1 |
| *Macaca thibetana* | Tibetan macaque | HM468437.1 |
| *Alouatta belzebul* | Red-handed howler monkey | GU382635.1 |
| *Alouatta sara* | Bolivian red howler monkey | AY843511.1 |
| *Aotus azarae* | Southern owl monkey | GU382632.1 |
| *Aotus trivirgatus* | Night monkey | AY740621.1 |
| *Saimiri sciureus* | Common squirrel monkey | AY843517.1 |
| *Saimiri boliviensis* | Black-capped squirrel monkey | AAW72442.1 |
| *Saimiri ustus* | Golden-backed squirrel monkey | GU382663.1 |
| *Callithrix jacchus* | Callithrix jacchus | XP_002754950.1 |
| *Callithrix kuhlii* | Wied's black-tufted-ear marmoset | GU382649.1 |
| *Callithrix penicillata* | Black-pencilled marmoset | GU382650.1 |
| *Callithrix geoffroyi* | Geoffroy's marmoset | GU382643.1 |
| *Mico argentatus* | Silvery marmoset | GU382640.1 |
| *Mico emiliae* | Snethlage's marmoset | GU382641.1 |
| *Mico humeralifer* | Tassel-eared marmoset | GU382644.1 |
| *Leontopithecus chrysopygus* | Golden-rumped lion tamarin | GU382655.1 |
| *Leontopithecus rosalia* | Golden lion tamarin | GU382657.1 |
| *Saguinus bicolor* | Brazilian bare-faced tamarin | GU382659.1 |
| *Saguinus imperator* | Emperor tamarin | GU382660.1 |
| *Saguinus labiatus* | Red-chested mustached tamarin | AAV91989.1 |
| *Saguinus mystax* | Moustached tamarin | GU382661.1 |
| *Saguinus oedipus* | Cotton-top tamarin | DQ229285.1 |
| *Pithecia irrorata* | Gray's bald-faced Saki | GU382664.1 |
| *Pithecia pithecia* | White-faced saki | AY843515.1 |
| *Callicebus donacophilus* | Bolivian titi | AY843519.1 |
| *Callicebus moloch* | Red-bellied titi | EU124690.1 |
